# Pure White Cell Aplasia and Immune Thrombocytopenia after Thymoma Resection: A Case Report and Review of the Literature

**DOI:** 10.1155/2022/8271069

**Published:** 2022-03-22

**Authors:** Michael Youssef, Tyler W. Stratton, Reid C. Gallant, Christine Young, Daniel Y. Li, Siavash Piran

**Affiliations:** ^1^Faculty of Medicine, University of Toronto, Toronto, Canada; ^2^Schulich School of Medicine & Dentistry, Western University, London, ON, Canada; ^3^Department of Pharmacy, Trillium Health Partners, Mississauga, Ontario, Canada; ^4^Department of Laboratory Medicine and Pathobiology, University of Toronto, Toronto, Canada; ^5^Department of Medicine, Division of Hematology, University of Toronto, Toronto, Canada

## Abstract

We report a case of pure white cell aplasia (PWCA) postthymoma resection in a 74-year-old male presenting with a 2-week history of fevers, night sweats, and severe febrile neutropenia. His pure white cell aplasia was treated with intravenous immunoglobulin (IVIg), granulocyte colony-stimulating factor (G-CSF), prednisone, and cyclosporine with a mixed response. He also developed immune thrombocytopenia, which responded well to a short course of eltrombopag. With continued cyclosporine treatment, his platelet counts were stable after stopping eltrombopag. The patient's cyclosporine treatment was complicated by renal failure, resulting in cessation of cyclosporine. His PWCA and immune thrombocytopenia significantly worsened after stopping cyclosporine, and unfortunately, he died from multiorgan failure and sepsis.

## 1. Introduction

Thymoma is the most common neoplasm of the thymus and comprises 20% of mediastinal neoplasms, with an incidence in the US of 0.13 per 100,000 person-years [1, 2]. Patients with thymoma are commonly asymptomatic, although they may present with anterior mediastinal mass symptoms such as chest pain, dyspnea, and cough. Thymomas are classified histologically according to WHO classification of thymic tumours. This system classifies thymomas on the histological appearance of neoplastic cells into the following categories: spindle (type A), dendritic or stellate (type B1-3), or a combined appearance with both spindle and dendritic neoplastic cells (type AB). Type B thymomas are further subdivided into B1, B2, and B3 by evaluating the relative abundance of epithelial cells and lymphocytes [3]. Thymoma management includes surgical resection, radiation, or other systemic therapies e.g. chemotherapy [4, 5].

Thymomas are associated with various immune-mediated para-neoplastic syndromes like myasthenia gravis, Good's syndrome, and/or pure red cell aplasia [4, 6]. In contrast, PWCA is a rare manifestation that can present in patients with thymoma. In PWCA, myelopoiesis is absent or strongly inhibited, while erythropoiesis and megakaryocytopoiesis remain relatively normal [7, 8]. This presents in the form of severe neutropenia, which can thereby result in recurrent life-threatening infections. Many therapies have been used for the treatment of PWCA with varying successes including corticosteroids, cyclosporine, cyclophosphamide, alemtuzumab, G-CSF, high dose IVIg, or plasmapheresis [8–12]. Surgical removal of thymoma can also help eliminate PWCA in some cases [7, 13].

## 2. Case Presentation

A 74-year-old male was admitted to our hospital with severe febrile neutropenia and the Hematology service was consulted. His past medical history was significant for hypertension, obesity, dyslipidemia, type 2-diabetes, and venous stasis. His home medications included metformin and atorvastatin.

**Figure 1:**
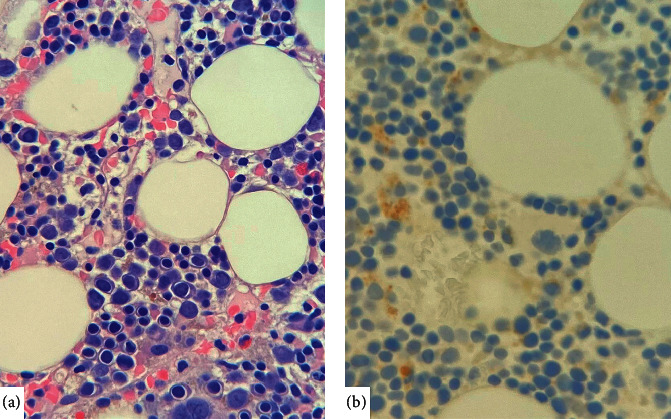
Granulocytic hypoplasia. Bone marrow core biopsy shows complete absence of granulocytic precursor elements on Hematoxylin/eosin staining (a) and confirmed by immunoperoxidase staining for myeloperoxidase (b). (40x objective).

The patient had a mediastinal mass resected, which was found to be a Type B1 thymoma. Neutrophil counts prior to surgery were found to be normal (Table 1). The patient was discharged 4 days later and then returned to hospital with a history of fevers and night sweats. On presentation, he was febrile at 39.7°C and tachycardic with a pulse of 110. He had an undetectable neutrophil count of <0.1 × 10^9^/L and was started on broad-spectrum antibiotics (Table 1). A bone marrow aspirate and biopsy showed granulocytic hypoplasia (Figure 1) in keeping with a diagnosis of PWCA postthymoma resection. A significantly reduced M: E ratio and mild megakaryocytic hyperplasia were also seen on bone marrow biopsy.

Based on laboratory investigations, the patient did not appear to have Good's Syndrome. At the onset of neutropenia, immunoglobulins were normal: IgA, 2.67 g/L; IgG, 12.90 g/L; IgM, 0.90 g/L. Peripheral blood flow cytometry demonstrated a normal number of T cells in peripheral blood with a CD4: CD8 ratio of 1 and normal CD19+ B cell population. Additionally, electromyography (EMG) did not show evidence of myasthenia gravis and serology for HIV, HBV, HCV, and TB was negative.

**Figure 2:**
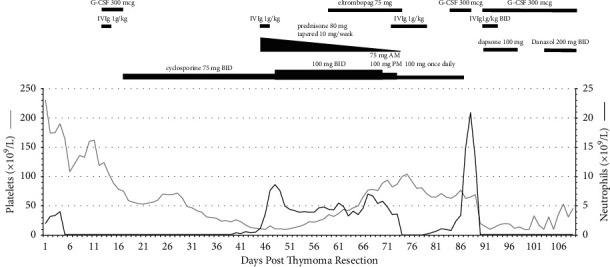
Trends in neutrophil and platelet counts following thymoma resection.

The patient was readmitted for a second time to the hospital with febrile neutropenia. He received IVIg 1 g/kg for 2 days as well as G-CSF 300 ug subcutaneously for 5 days (Figure 2). However, there was no response and the neutrophil count remained <0.1 × 10^9^/L. Therefore, the patient was started on cyclosporine 75 mg BID (Figure 2). Due to persistent severe neutropenia (<0.1 × 10^9^/L), the patient was started on prednisone 1 mg/kg once daily. The trough cyclosporine level was 147 ug/L 4 days after starting cyclosporine. The dose of cyclosporine was then increased to 100 mg BID (Figure 2). We used a target cyclosporine level of 200 to 400 ug/L. A second course of G-CSF was started. His neutrophil count normalized, however he developed severe thrombocytopenia (Figure 2).

The patient's thrombocytopenia was also thought to be immune-mediated and therefore he was started on eltrombopag 75 mg once daily (Figure 2). He responded well to eltrombopag, reaching a platelet count of 50 × 10^9^/L. The patient's prednisone was tapered off. Eltrombopag was also tapered off and the patient was discharged home on only cyclosporine.

Despite cyclosporine treatment, he was readmitted to the hospital for the third time with febrile neutropenia undetectable neutrophil count in keeping with relapsed PWCA. He was then started on IVIg 1 g/kg for two days and supportive G-CSF with good response and his neutrophil count normalized (Figure 2). He was discharged home on cyclosporine 100 mg BID.

Although his cyclosporine level was in the target range of 200 to 400 ug/L, the patient developed worsening acute kidney injury. The cyclosporine dose was further reduced to 50 mg BID. However, his creatinine continued to rise despite a 50% reduction in his cyclosporine dose. At this point, the patient had been on cyclosporine for 5 months and a response was seen in his counts. As such, cyclosporine was discontinued due to worsening kidney function. Unfortunately, the patient's kidney function did not normalize and his counts dropped. He was started on dapsone 100 mg once daily followed by danazol 200 mg BID (Figure 2), which are both used for the management of refractory immune thrombocytopenia [14]. Dapsone and danazol were used as immunosuppressive therapies to target his immune cytopenias. However, his cytopenias continued to worsen and he was admitted to the hospital with weakness and febrile neutropenia. Despite treatment with dapsone, danazol, IVIg, and G-CSF his PWCA did not respond to any of these therapies and he died from sepsis and multiorgan failure.

## 3. Discussion

Thymomas are rare epithelial tumors that commonly present with paraneoplastic syndromes, with up to 50% of thymoma patients presenting with associated immunodeficiencies and autoimmune phenomena [15]. A recent systematic review reports estimates of common paraneoplastic syndromes in thymoma patients including myasthenia gravis (63%), pure red cell aplasia (7.7%) and hypogammaglobulinemia referred to as Good's syndrome (6%) [16]. However, PWCA is a rare disorder with only a few existing case reports. The etiology underlying PWCA remains elusive, although an autoimmune origin with dysregulated production of cytokines and antibody-mediated destruction of myelomonocytic precursor cells is speculated [7]. The severe neutropenia in these patients increases the risk of recurrent life-threatening infections and can even result in mortality. Our patient did not have any prior history of myasthenia gravis and his EMG testing was negative for any neuromuscular disorder. His blood work showed only PWCA and immune thrombocytopenia, with no evidence of hypogammaglobulinemia. Although most previous case reports have described PWCA at the time of thymoma diagnosis, our patient's first evidence of neutropenia first developed 2-weeks postthymectomy [17]. Here, we have reviewed and summarized several case reports for PWCA associated with thymoma (Table 2). However, to our knowledge, this is the first report of immune dysregulation with PWCA and immune thrombocytopenia post thymoma resection.

Each of the cases reported in Table 2 had the presence of thymoma and PWCA similar to our case presented here. In keeping with previous studies, the mean age was 62 (range of 36 to 76 years) with nine females and nine males [27]. Of the 18 cases examined, only 7 reported comorbidities including myasthenia gravis, autoimmune thyroiditis, type 1 diabetes, inflammatory bowel disease, clotting factor deficiencies or even hematological cancers like leukemia. In our case presented here, the patient had many more comorbid conditions than previous case reports.

The thymoma histology of cases examined in Table 2 varied widely, with type A being the most common. In our case report, the patient's thymoma type was classified as type B1. The WHO thymoma classification categories carry prognostic significance for patients. Reports show that those diagnosed with Type A and Type AB have 100% and 90% survival after 15 years, respectively. Types B1, B2, and B3 have been associated with 90%, 60% and 40% survival after 20 years, respectively [28]. However these estimates may differ in cases where patients with thymoma also develop PWCA.

A variety of different management options have been attempted in patients with PWCA and thymoma. A common approach involves immunosuppression with treatments including IVIg, cyclosporine, cyclophosphamide, azathioprine, corticosteroids, and mycophenolate mofetil (Table 1). Patients also commonly receive antibiotics, antifungals, and antivirals for comorbid infections. Additionally, G-CSF and alemtuzumab are often used to stimulate granulocyte or lymphocyte counts. Lastly, thymectomy and plasmapheresis have also been shown to raise granulocyte levels [8, 21].

Of the 18 cases with PWCA and thymoma examined here (including our case), 7 patients died. With our patient, treatment with IVIg, G-CSF, prednisone, and cyclosporine were unsuccessful. He ultimately died from sepsis and multiorgan failure, which illustrates the poor prognosis of PWCA. His age, comorbidities, and immune thrombocytopenia may have decreased his chance of survival. Despite this patient's poor clinical course, treatment with cyclosporine as a first-line therapy with concomitant use of G-CSF is still recommended. These two therapies were shown to be effective in previous case reports and initially restored our patient's granulocytic counts [29]. In the future, consideration should also be given to alternative immunosuppressive therapies such as rituximab, azathioprine or alemtuzumab, all of which were shown to have some effectiveness in treating PWCA [29, 30]. We also recommend long-term follow-up with thymoma patients given the high risk of relapse of PWCA as with our patient.

## 4. Conclusion

Our case report identifies a complex case of PWCA and immune thrombocytopenia postthymoma resection. This patient was treated with IVIg, G-CSF, prednisone, and cyclosporine with a mixed response. Our review of the literature and the current case highlights the high mortality rate observed in patients with PWCA. Future studies are needed to compare the clinical course of thymoma patients with and without PWCA to better understand the burden of disease and the utility of therapeutic intervention in management of PWCA.

## Figures and Tables

**Table 1:** Complete blood count values prethymectomy, postthymectomy, and at the onset of thrombocytopenia.

Value	Prethymectomy	Postthymectomy	At onset of thrombocytopenia	Ref. range
WBC	11.5^*∗*^	3.4^*∗*^	3.1^*∗*^	4.0–11.0 × 10⁹/L
RBC	4.50	4.28	2.46^*∗*^	4.0–5.5 × 10^12^/L
HGB	118^*∗*^	114^*∗*^	76^*∗*^	135–170 g/L
HCT	0.364^*∗*^	0.352^*∗*^	0.227^*∗*^	0.380–0.500 L/L
MCV	80.9	82.2	92.3	80–100 fL
MCH	26.2	26.6	30.9	25–34 pg
MCHC	324	324	335	300–365 g/L
RDW	16.9^*∗*^	16.7^*∗*^	21.1^*∗*^	11.5–15.5%
PLT	231	164	11^*∗*^	150–400 × 10⁹/L
Neutrophils	2.0	<0.1^*∗*^	1.2^*∗*^	2.0–7.5 × 10⁹/L

WBC: white blood cell; RBC: red blood cell; HGB: hemoglobin; HCT: hematocrit; MCV: mean corpuscular volume; MCH: mean corpuscular hemoglobin; MCHC: mean corpuscular hemoglobin concentration; RDW: red blood cell distribution width; PLT: platelet count.

**Table 2:** Literature review of previous case reports for PWCA associated with thymoma. IVIg: Intravenous Immunoglobulins, G-CSF: Granulocyte Colony-Stimulating Factor.

Study	Ackland et al. 1988 [18]	Akinosoglou et al. 2014 [20]	Al-mohareb et al. 1992 [22]	Alvares et al. 2004 [21]	Degos et al. 1982 [8]	Desai et al. 2013 [23]	Fumeaux et al. 2003 [13]	Jethava et al. 2011 [24]
Patient age (gender)	70 (female)	70 (female)	70 (male)	59 (male)	52 (female)	73 (male)	76 (female)	45 (male)
Patient presentation	Neutropenia Pharyngeal candidiasis Hypogammaglobulinemia Sepsis (*Pseudomonas aeruginosa*) Recurrent upper respiratory infections	Fatigue Persistent fevers Disseminated Cryptococcosis	Fatigue Sweating Chest pain Productive cough Intermittent fevers	Fever Fatigue Aphthous oral ulcers	Mild anemia Agranulocytosis Hypogammaglobulinemia Severe recurrent infections (bacterial, mycotic, and parasitic)	Vomiting Dyspnea Dysphagia Atrial Fibrillation Abdominal Pain Oral candidiasis Productive cough	Fever Fatigue Diarrhea Weakness Weight loss Odynophagia Hyperglycemia	Hypoxia Dyspnea Oral ulcers Tachycardia Febrile neutropenia Factor XI deficiency Respiratory infection with lung nodules (*Aspergillus fumigatus*)
Comorbidities	Myasthenia gravis	Obesity Dyslipidemia	—	—	—	—	Autoimmune thyroiditis type 1 diabetes	Factor XI deficiency
Histology of thymoma (type)	Metastatic spindle cell thymoma	Spindle cell thymoma (type A)	Spindle cell thymoma (type A)	Spindle cell thymoma (type A)	Spindle cell thymoma (type A)	Thymic carcinoma (type C)	Malignant cortical thymoma (type B2)	Thymoma (type AB)
Management (chronological)	Broad spectrum antibiotics Pyridostigmine IVIg 0.4 g/kg/day	Broad spectrum antibiotics Amphotericin B G-CSF IVIg dexamethasone Acyclovir Fluconazole	Broad spectrum antibiotics Antifungal agents Antiviral medications	Broad spectrum antibiotics Antifungal agents G-CSF (5 *μ*g/kg/day) thymectomy Plasmapheresis Campath-1H 100 mg Cyclosporin 150 mg bid Mycophenolate mofetil 500 mg bid	Thymectomy Plasmapheresis Prednisone 1.5 mg/kg/day Cyclophosphamide 2 mg/kg/day	—	IVIg 15 g/day G-CSF thymectomy Cyclosporin a 3 mg/kg/d (in two doses Prednisone 1 mg/kg/d	Broad spectrum IV antibiotics & antifungals IVIg 0.4 g/kg/d G-CSF cyclosporine A Thymectomy
Mortality outcome	Died	Survived	Died	Survived	Survived	n/a	Survived	Survived
Comments					Plasmapheresis Appeared to be the only effective treatment		IVIg and G-CSF were only effective when given with methylprednisone after thymectomy	

Study	Kobayashi et al. 2019 [25]	Mathieson et al. 1990 [19]	Okusu et al. 2016 [26]	Oyenuga et al. 2021 [17]	Uy et al. 2019 [7]	Yip et al. 1996 (1/2) [12]	Yip et al. 1996 (2/2) [12]	
Patient age (gender)	63 (male)	36 (female)	72 (male)	64 (male)	65 (female)	51 (male)	52 (female)	
Patient presentation	Febrile neutropenia Hypogammaglobulinemia	Diarrhea Gingivitis Oral ulcers Abdominal pain Febrile neutropenia	Sore throat Myocarditis Oral candidiasis Febrile neutropenia Atrioventricular block Hypogammaglobulinemia Respiratory infection (*Pseudomonas aeruginosa*)	nasal congestion Cough Orthopnea Diverticulitis Gram-negative septic shock Recurrent colitis Hypogamma-globulinemia	Night sweats Oral candidiasis Febrile neutropenia Truncal morbilliform rash Hypogammaglobulinemia Lung nodules (likely respiratory fungal infections)	Night sweats Febrile neutropenia Hypogamma-globulinemia Upper respiratory tract infections	Lethargy Oral ulcers Sore throat Weight loss Oral candidiasis Febrile neutropenia	
Comorbidities	—	Myasthenia gravis	—	Inflammatory bowel disease	—	—	—	
Histology of thymoma (type)	Spindle cell thymoma (type A)	Thymoma (lymphoepithelial type)	Thymoma (type 2B)	Thymoma (type 2B)	Thymoma (mixed type a and B2)	Spindle cell thymoma (type A)	Spindle cell thymoma (type A)	
Management (chronological)	Oral garenoxacin (by primary care physician) G-CSF 5 *μ*g/kg/d cyclosporin a 150 mg/d Thymectomy	Plasmapheresis Prednisolone 70 mg every other day Azathioprine 2.5 mg/kg/d	Antibiotic therapy IVIg	Thymectomy IVIg	IVIg G-CSF thymectomy Cyclosporin (target blood level 200–400 ng/mL)	Broad spectrum antibiotics Prednisone 100 mg/d G-CSF 150–600 *μ*g/d IVIg	GM-CSF 5–10 *μ*g/kg/d methylprednisolone 1 g/d Plasmapheresis Thymectomy IVIg 500 mg/kg/d cyclophosphamide 100 mg/d	
Mortality outcome	Survived	Survived	Died	Survived	Survived	Survived	Died	
Comments		Prednisone and azathioprine were the successful treatments	No specific treatment for PWCA since the patient developed AV block and shock			Thymectomy had no effect		

## Data Availability

Data supporting findings of this study are available from the corresponding author upon request.
